# Natalizumab in the pediatric MS population: results of the Italian registry

**DOI:** 10.1186/s12883-015-0433-y

**Published:** 2015-09-25

**Authors:** Angelo Ghezzi, Lucia Moiola, Carlo Pozzilli, Vincenzo Brescia-Morra, Paolo Gallo, Luigi Maria Edoardo Grimaldi, Massimo Filippi, Giancarlo Comi G., the MS Study Group-Italian Society of Neurology

**Affiliations:** Multiple Sclerosis Study Center, Hospital of Gallarate, Via Pastori 4, 21013 Gallarate, Italy; Department of Neurology, Hospital S. Raffaele, Milan, Italy; S. Andrea Multiple Sclerosis Center, University of Rome La Sapienza, Rome, Italy; MS Centre, Neurosciences, Reproductive and Odontostomatological Sciences Department, Federico II University of Naples, Naples, Italy; Department of Neurology, University of Padua, Padua, Italy; U.O.C. Neurologia, Fondazione Istituto San Raffaele “G.Giglio”, Cefalù, Italy; Neuroimaging Research Unit, Institute of Experimental Neurology, Division of Neuroscience, San Raffaele Scientific Institute, Vita-Salute San Raffaele University, Milan, Italy

## Abstract

**Background:**

Natalizumab is a promising option for pediatric multiple sclerosis (MS) patients with active evolution and a poor response to Interferon-beta or Glatiramer Acetate. However, no data are available in large cohorts of patients and after a long-term follow up. Our study was planned to shed lights on this topic.

**Methods:**

A registry was established in 2007 in Italy to collect MS cases treated with Natalizumab (NA) before 18 years of age.

**Results:**

101 patients were included (69 females), mean age of MS onset 12.9 ± 2.7 years, mean age at NA initiation 14.7 ± 2.4 years. Mean treatment duration was 34.2 ± 18.3 months.

During NA treatment, a total of 15 relapses were recorded in 9 patients, annualized relapse rate was 2.3 ± 1.0 in the year prior to NA and decreased to 0.1 ± 0.3 (*p* < 0.001) at last NA infusion.

Mean Expanded Disability Status Scale (EDSS) decreased from 2.6 ± 1.3 at initiation of NA to 1.8 ± 1.2 at the time of last visit (*p* < 0.001). At brain MRI, new T2 or Gd enhancing lesions were observed in 10/91 patients after 6 months, 6/87 after 12 months, 2/61 after 18 months, 2/68 after 24 months, 3/62 after 30 months, and 5/43 at longer follow up. At the time of last observation, 58 % of patients were free from clinical (relapses/increased EDSS) and/or MRI activity (new T2 or gadolinium-enhancing lesions). No relevant adverse events were recorded.

**Discussion:**

NA was safe, well tolerated and very efficacious in the large majority of patients. Our data support the use of this medication in subjects with pediatric MS and an aggressive course.

**Conclusions:**

A relevant reduction of relapse rate and EDSS was observed during NA treatment, compared to pre-treatment period. No evidence of disease activity (NEDA) occurred in 58 % of cases.

## Background

Natalizumab (NA) is approved for the treatment of the relapsing form of multiple sclerosis (MS) for patients with inadequate response to first line treatments or with severe and active MS. First line medications, namely interferon beta (IFNB) and glatiramer acetate (GA), are the standard care of patients with pediatric with MS (ped-MS) [1–3], however about 30 % of them are partial or non-responder to first line treatments requiring a shift to other disease modifying treatments (DMT) [4]. Some observational studies have shown that NA consistently reduces disease activity in patients with ped-MS [5–10]. No relevant adverse events have been reported up to now, the major concern remaining the possible occurrence of progressive multifocal leukoencephalopathy (PML) [11, 12]. This risk can now be evaluated by assessing the presence and the level of anti-JCV antibodies [13, 14].

NA is considered as a promising option for ped-MS patients with active MS and a poor response to IFNB and GA, with the limitation that data are not available in large cohorts of patients and after a long-term follow up [15]. We conducted this study to provide further pieces of information on this topic.

## Methods

A registry was established in 2007 in Italy, within the MS Study Group of the Italian Society of Neurology, with the objective to collect all cases treated with NA before the age of 18 years and mandatorily included in the Italian Medicine Agency (AIFA) registry [5, 6]. Patients were eligible if they received at least one NA administration and had at least 3 months of observation. Details on study design (inclusion criteria, definition of relapse, neurological evaluation, NA administration, have been previously described [5, 6].

In the majority of cases brain MRI was performed every 6 months. The clinical records of 55 patients previously included in the database [6] have been updated up the June 2014 when other 46 new cases had been included.

Anti-JCV antibodies in patients’ serum were assessed since the test *Stratify JCVTM* (performed at Unilabs, Copenhagen, Denmark) became available in Italy (March 2011).

The study was conducted in accordance with the International Conference on Harmonisation Guidelines of Good Clinical Practice and the Declaration of Helsinki. The protocol received approval from the ethical committee of the coordinating center (Ospedale di Gallarate, Sept. 2007) [5, 6]. Written informed consent was obtained from each patient participating in the study and her or his parents.

## Results

One hundred-one patients were included (69 females), mean age (SD) of MS onset 12.9 ± 2.7 years, mean age (SD) at NA initiation 14.7 ± 2.4 years, mean treatment duration (SD) 34.2 ± 18.3 months. NA was administered at a standard dose of 300 mg every 28 days. Patients demographic data are reported in Table 1.

**Table 1:** Demographic and baseline clinical characteristics

	Mean (SD)
Age at MS onset (years)	12.9 (2.7)
Pre-NA disease duration (months)	25.6 (23.3)
Age at NA initiation (years)	14.7 (2.4)
Weight (Kg)	62.0 (13.3)
Height (cm)	163.5 (12.3)
Number of relapses prior NA initiation	4.0 (2.2)
Number of relapses in the year prior NA initiation	2.3 (1.3)
Number of Gd + MRI lesions prior NA initiation	3.3 (4.4)
EDSS at NA initiation	2.6 (1.3)

**Table 2:** Clinical and laboratory adverse events

Clinical adverse events	Number of subjects
Headache	13
Upper respiratory disorders	9
Vertigo	7
Gastrointestinal disorders	5
Edema, itching, dermatitis	6
Herpes zoster	4
Restlessness	2
Urinary tract infections	3
Fatigue	4
Infections	2
Menstrual disorders	2
Ovarian cyst	1
Sacral cyst	1
Muscular pain	1
Weight loss	3
Depression	1
Hypertension	1
Tachycardia	1
Total	66
Laboratory abnormalities	Number of subjects
Increased white blood cells	9
Increased bilirubin	2
Increased ALT/AST	2
Proteinuria	1
Anemia	1
Total	15

Sixty-six patients had been treated previously with immunosuppressants/ immunomodulators: 58 with IFNB, 9 with GA, 7 with immunosuppressants.

A total of 15 relapses were recorded in 9 patients during the whole treatment period; 8 of them occurred in the first year of treatment. The mean annualized relapse rate (SD) was 2.3 ± 1.3 in the year prior to NA and decreased to 0.1 ± 0.3 at the time of the last NA infusion (*p* < 0.001).

Mean EDSS (SD) decreased from 2.6 ± 1.3 at NA initiation to 1.8 ± 1.2 at the time of last visit (*p* < 0.001). EDSS remained stable in 27 cases, decreased by at least 0.5 point in 22 cases, by 1 point or more in 46 patients, and increased by 0.5 point in5 patients and by > 0.5 in one (with increased T2 lesions at month 12).

MRI was performed within 1 month prior to starting NA in 93 patients, showing a mean number of 3.3 ± 4.4 Gd-enhancing lesions in 77 of them (82.8 %). New lesions on T2-weighted sequences or Gd-enhancing lesions were observed in 10/91 (10.9 %) patients at 6 months, 6/87 (6.9 %) patients at 12 months (including one patient tested at month 9), 2/61 (3.3 %) patients at 18 months, 2/68 (2.9 %) patients at 24 months, 3/62 (4.8 %) after 30 months, and 5/43 (10.6 %) with a longer follow up.

**Fig. 1:**
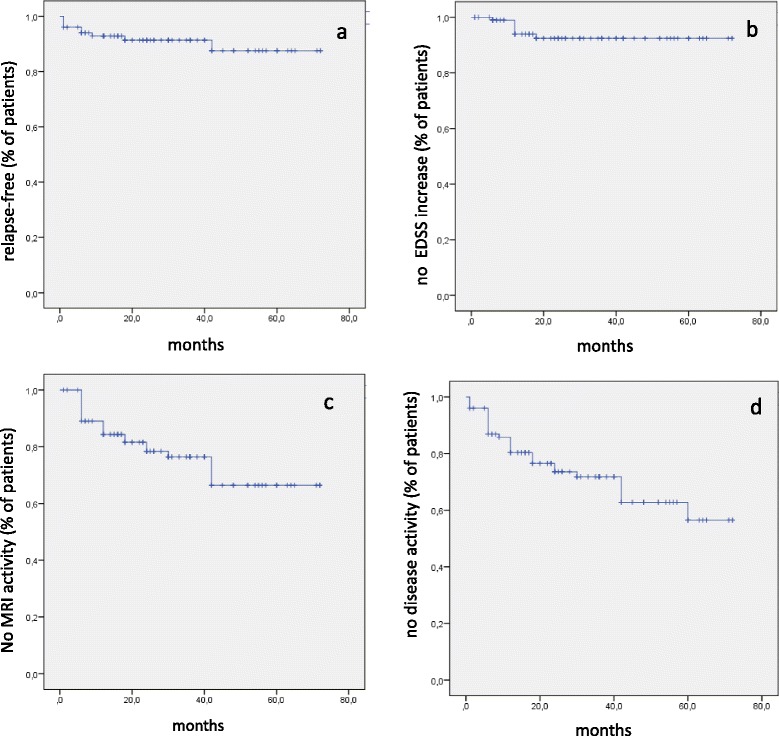
Kaplan-Meyer curves reporting the frequency of patients free from relapses (**a**), disease progression, defined by an increase of EDSS score at the last observation (**b**), MRI activity (**c**), and disease activity (no clinical and/or MRI activity) (**d**)

Survival curves were used to calculate the frequency of patients free from clinical (no relapses, no EDSS increase) and/or MRI activity (no new T2, no Gd-enhancing lesions). At the time of the last observation,  58 % of cases showed no evidence of disease (clinical and MRI) activity (NEDA) (Fig. 1)

Serum anti-JCV antibodies were assessed in 100 patients, including 3 subjects not tested in our previous study; one discontinued therapy before the test became available. At baseline, the test was positive in 43/100 patients. During the follow up period the test was repeated in 45 subjects: 20 subjects remained negative, 18 subjects remained positive, 5 subjects shifted from the negative to the positive status, 2 vice versa (ratio not available at the time of the first assessment). The anti-JCV antibody index was determined in 21 subjects (<0.9 in 4 subjects; between 0.9 and 1.5 in 2 subjects; > 1.5 in 15 subjects).

A total of 66 clinical adverse events were observed in 36/101 patients (Table 2). All of them were mild, did not cause drug discontinuation and, if necessary, were treated according to normal standard of medical care. Mild hematological abnormalities (below toxicity level 1) were observed in 15/101 cases (Table 2): they resolved spontaneously and no intervention was required. Five patients were treated with NA before 10 years of age: transitory restlessness was recorded in one patient and transitory increase of WBC in another one.

At the time of the last update, in June 2014, NA was discontinued in 19 cases for various reasons: 2 for MS progression (after 9 and 30 infusions), 6 for anti-JCV Ab positivity (4 of them shifted to other DMT), 6 for parent/patient decision, other 4 patients suspended NA or shifted to other therapies but returned to NA after disease reactivation; one was lost a follow up. The mean time of withdrawal (SD) was 18.1 ± 14.2 months, during this period 10 patients relapsed (relapse rate 1.3 ± 1.2), 2 patients continued to worsen, 4 patients presented MRI reactivation, only 3 remained stable after 12–36 months.

Clinical data of patients included in our previous study (5) were updated after two additional years of follow up (mean follow up 47.2 ± 17.9 months). At the time of the last NA administration, after a mean of 42.9 ± 15.5 infusions, the mean EDSS was 1.9 ± 1.2 (mean initial score 2.7 ± 1.2), and only 4 patients relapsed, for a total of 5 relapses. Thirty-four patients continued to be free from clinical (neither relapses nor disability progression as measured by EDSS) and MRI activity (no new lesions on T2-weighted sequences and no Gd-enhancing lesions).

## Discussion

The Italian registry of pediatric MS patients treated with NA was created seven years ago, within the Italian Multiple Sclerosis Study Group of the Italian Society of Neurology, to collect data on long-term safety and effectiveness of this medication in subjects before 18 years of age. Initially the use of NA was off-label and required the authorization by the Italian Agency of Drugs (AIFA) for each patient. After 2012, NA has been authorized in Italy for pediatric MS, although restricted to patients with diagnosis of definite MS and less than 18 years if they presented at least two relapses in the previous year and an increase of the EDSS score. One-hundred-one patients have been included in the registry up to now, all with an active form of MS: the mean relapse rate was 2.3 and the mean number of Gd enhancing lesions at brain MRI was 3.3 in the year prior NA initiation. To our knowledge, this is the largest study of ped-MS patients treated with NA, and with the longest follow up published so far. Eight pediatric MS patients have been described by Arnal Garcia et al. after 1 year of NA treatment [10], and 20 by Kornek et al after 20 months of follow up [9], finding a relevant reduction of relapse rate (respectively from 3.9 to 0.4 and from 3.7 to 0.4). No relapses and no MRI activity was found by Yeh & Weinstock-Guttman in 20 out of 24 cases with a mean age of 14 years, treated with NA for a mean of 1.5 years [8].

In the present study we could confirm the good safety profile of NA in the pediatric population, with a frequency of adverse events similar to that reported in our previous study: they were mild in all cases, and, if necessary, they were treated according to usual medical procedures; in no patient NA had to be discontinued. The follow up of two additional years of the cohort of 55 subjects published in 2013 did not reveal the occurrence of other rare or unexpected events. Laboratory abnormalities were mild, confirming the good safety profile of NA in the pediatric population.

We could also confirm the excellent clinical response of NA, demonstrated by the impressive reduction of relapses during the exposure to NA therapy. Only 9 patients developed relapses, for a total of 15 relapses, 8 of them recorded in in the first year of treatment. In the large majority of patients treatment with NA also induced an improvement of disability as measured by EDSS score, whereas only three patients worsened during the study. In two of them NA was stopped. Compared to the baseline score, EDSS was unchanged in 27 subjects, decreased by 0.5 or more in 68 subjects, increased by 0.5 or more in 6 subjects.

The favourable effect of NA was supported by MRI findings, showing a consistent reduction of MRI activity (defined by new T2-hyperintense or Gd enhancing lesions) in the large majority of patients. Using NEDA definition (no evidence of disease activity), 58  % of the subjects achieved it at the time of last observation.

Tests to detect anti-JCV antibodies are currently available, allowing a fair and careful evaluation of the risk of PML associated to NA exposure. This risk is higher in anti-JCV antibody positive individuals and correlates with the anti-JCV antibody levels, especially after the second year of treatment [13, 14]. A previous exposure to immunosuppressants is also correlated to an increased risk of PML [16]. The frequency of patients positive for anti-JCV antibodies at baseline was 43 % in our cohort, lower than the 57 % prevalence observed in an Italian cohort of adult patients [17] and similar to that observed in another cohort of MS pediatric patients [9]. Although this figure is reassuring relatively to the potential risk of PML, the possibility of seroconversion over time must be considered: it occurred in 5 subjects whereas surprisingly 2 patients shifted from the positive to the negative status. This latter observation suggests that the Stratify test should be repeated cyclically in NA-treated MS patients to obtain clinically useful updates of their PML risk.

Trials are currently ongoing in the pediatric MS populations evaluating the effect of new medications (fingolimod, BG12, teriflunomide). Our experience shows that also the implementation of a clinical registry is an important tool to collect important information on long-term data about the efficacy and tolerability of new medications in ped-MS.

## Conclusions

NAT was used in a cohort of patients before 18 years of age with an active form of MS, the majority of them with a poor response to first line treatments: this medication a consistently reduced relapse rate and disability. No evidence of disease activity (no relapses, no increase of the EDSS score, no new T2 lesions and no gadolinium enhancing lesions on brain MRI) was observed in 58 % of cases. NAT can be strongly considered as an option for patients with active ped-MS, particularly in cases negative for anti-JCV antibodies. No relevant adverse events were recorded and the medication was well tolerated.

## References

[1] Waldman A, Ghezzi A, Bar-Or A, Mikaeloff Y, Tardieu M, Banwell B (2014). Multiple sclerosis in children: an update on clinical diagnosis, therapeutic strategies, and research. Lancet Neurol..

[2] Chitnis T, Tardieu M, Amato MP, Banwell B, Bar-Or A, Ghezzi A (2013). International Pediatric MS Study Group Clinical Trials Summit: meeting report. Neurology..

[3] Ghezzi A, Banwell B, Boyko A, Amato MP, Anlar B, Blinkenberg M (2010). The management of multiple sclerosis in children: a European view. Mult Scler.

[4] Yeh EA, Waubant E, Krupp LB, Ness J, Chitnis T, Kuntz N (2011). Therapies in Pediatric Patients With Refractory Multiple Sclerosis. Arch Neurol.

[5] Ghezzi A, Pozzilli C, Grimaldi LM, Brescia Morra V, Bortolon F, Capra R (2010). Safety and efficacy of natalizumab in children with multiple sclerosis. Neurology..

[6] Ghezzi A, Pozzilli C, Grimaldi LM, Moiola L, Brescia-Morra V, Lugaresi A (2013). Natalizumab in pediatric multiple sclerosis: results of a cohort of 55 cases. Mult Scler.

[7] Huppke P, Stark W, Zürcher C, Huppke B, Brück W, Gärtner J (2008). Natalizumab use in pediatric multiple sclerosis. Arch Neurol..

[8] Yeh EA, Weinstock-Guttman B (2010). Natalizumab in pediatric multiple sclerosis patients. Ther Adv Neurol Disord..

[9] Kornek B, Aboul-Enein F, Rostasy K, Milos RI, Steiner I, Penzien J (2013). Natalizumab therapy for highly active pediatric multiple sclerosis. JAMA Neurol.

[10] Arnal-Garcia C, García-Montero MR, Málaga I, Millán-Pascual J, Oliva-Nacarino P, Ramió-Torrentà L (2013). Natalizumab use in pediatric patients with relapsing-remitting multiple sclerosis. Eur J Paediatr Neurol..

[11] Butzkueven H, Kappos L, Pellegrini F, Trojano M, Wiendl H, Patel RN (2014). TYSABRI Observational Program (TOP) Investigators. Efficacy and safety of natalizumab in multiple sclerosis: interim observational programme results. J Neurol Neurosurg Psychiatry.

[12] Kappos L, Bates D, Edan G, Eraksoy M, Garcia-Merino A, Grigoriadis N (2011). Natalizumab treatment for multiple sclerosis: updated recommendations for patient selection and monitoring. Lancet Neurol..

[13] Gorelik L, Lerner M, Bixler S, Crossman M, Schlain B, Simon K (2010). Anti-JC virus antibodies: implications for PML risk stratification. Ann Neurol.

[14] Plavina T, Subramanyam M, Bloomgren G, Richman S, Pace A, Lee S (2014). Anti-JC virus antibody levels in serum or plasma further define risk of natalizumab-associated progressive multifocal leukoencephalopathy. Ann Neurol..

[15] Chitnis T, Tenembaum S, Banwell B, Krupp L, Pohl D, Rostasy K (2012). Consensus statement: evaluation of new and existing therapeutics for pediatric multiple sclerosis. Mult Scler.

[16] Sørensen PS, Bertolotto A, Edan G, Giovannoni G, Gold R, Havrdova E (2012). Risk stratification for progressive multifocal leukoencephalopathy in patients treated with natalizumab. Mult Scler.

[17] Moiola L, Sangalli F, Martinelli V (2011). Prevalence of anti-JCV antibodies in a cohort of natalizumab-treated multiple sclerosis patients from Italy. Neurology Suppl.

